# Neglect of Medical Evidence of Torture in Guantánamo Bay: A Case Series

**DOI:** 10.1371/journal.pmed.1001027

**Published:** 2011-04-26

**Authors:** Vincent Iacopino, Stephen N. Xenakis

**Affiliations:** 1 Physicians for Human Rights, Cambridge, Massachusetts, United States of America; 2 University of Minnesota School of Medicine, Minneapolis, Minnesota, United States of America; 3 Human Rights Center, University of California, Berkeley, Berkeley, California, United States of America; 4 Brigadier General (Retired), US Army, United States of America; University of Cape Town, South Africa

## Abstract

**Background:**

In the wake of the September 11, 2001 attacks on the US, the government authorized the use of “enhanced interrogation” techniques that were previously recognized as torture. While the complicity of US health professionals in the design and implementation of US torture practices has been documented, little is known about the role of health providers, assigned to the US Department of Defense (DoD) at the US Naval Station Guantánamo Bay, Cuba (GTMO), who should have been in a position to observe and document physical and psychological evidence of torture and ill treatment.

**Methods and Findings:**

We reviewed GTMO medical records and relevant case files (client affidavits, attorney–client notes and summaries, and legal affidavits of medical experts) of nine individuals for evidence of torture and ill treatment and documentation by medical personnel. In each of the nine cases, GTMO detainees alleged abusive interrogation methods that are consistent with torture as defined by the UN Convention Against Torture as well as the more restrictive US definition of torture that was operational at the time. The medical affidavits in each of the nine cases indicate that the specific allegations of torture and ill treatment are highly consistent with physical and psychological evidence documented in the medical records and evaluations by non-governmental medical experts. However, the medical personnel who treated the detainees at GTMO failed to inquire and/or document causes of the physical injuries and psychological symptoms they observed. Psychological symptoms were commonly attributed to “personality disorders” and “routine stressors of confinement.” Temporary psychotic symptoms and hallucinations did not prompt consideration of abusive treatment. Psychological assessments conducted by non-governmental medical experts revealed diagnostic criteria for current major depression and/or PTSD in all nine cases.

**Conclusion:**

The findings in these nine cases from GTMO indicate that medical doctors and mental health personnel assigned to the DoD neglected and/or concealed medical evidence of intentional harm.

*Please see later in the article for the Editors' Summary*

## Abstract 2

Vincent Iacopino and Stephen Xenakis review case records of nine individuals imprisoned at Guantánamo Bay which indicate that medical personnel assigned to the US Department of Defense neglected and/or concealed medical evidence of torture.

## Editors' Summary

**Background:**

Torture has been used throughout history for interrogation, coercion, and punishment. Ingenious methods have been devised to inflict severe physical or mental pain or suffering intentionally on an individual to obtain a confession or information, or to punish, intimidate, or coerce. Nowadays, torture is prohibited under international law and under the domestic law of most countries, and is considered to be a violation of human rights. Article 5 of the United Nations (UN) Universal Declaration of Human Rights, which was adopted in December 1948, states: “No one should be subjected to torture or to cruel, inhuman or degrading treatment or punishment.” Similarly, signatories of the Geneva Conventions, which provide protection for people who fall into enemy hands during conflicts, have agreed not to torture prisoners. Torture is also prohibited by the UN Convention against Torture and Other Cruel, Inhuman or Degrading Treatment or Punishment, which came into force in June 1987. Implementation of this Convention by participating states is monitored the UN Committee against Torture.

**Why Was This Study Done?:**

After the September 11, 2001 attacks on the United States, the US government redefined acts such as waterboarding (simulated drowning), sleep deprivation, and prolonged isolation as “safe, legal, ethical, and effective” “enhanced interrogation” techniques (EITs). These EITs were previously recognized as torture by the UN Committee against Torture. US health professionals are known to have been complicit in the design and implementation of EITs. For example, the US Central Intelligence Agency (CIA) and Department of Defense (DoD) designated “non-clinical” health professionals to monitor the use of EITs at the US detainee facility at the US Navy Base at Guantánamo Bay, Cuba (GTMO), and the active role of these individuals during interrogations has been documented. Much less is known, however, about the role of health professionals assigned to the DoD to provide medical and mental health care to the GTMO detainees. Specifically, it is not known whether these health professionals accurately documented physical and psychological evidence of torture and ill treatment among the detainees. In this case series, the researchers review GTMO medical records and case files of nine detainees for evidence of documentation of ill treatment and torture by medical personnel.

**What Did the Researchers Do and Find?:**

The researchers—non-governmental medical experts retained by legal representatives of GTMO detainees alleging torture and ill treatment—reviewed the medical records, client affidavits, attorney–client notes, and legal declarations of medical experts of nine GTMO detainees. In each case, the detainee alleged abusive interrogation methods consistent with torture as defined by the UN Convention against Torture. The researchers report that the medical affidavits for all the cases indicate that the allegations of torture and ill treatment were consistent with physical and psychological evidence of torture and ill treatment documented in the medical records and in evaluations by non-governmental experts. However, the medical personnel responsible for the detainees' routine medical and mental health care failed to inquire about and/or document the causes of the physical injuries and psychological symptoms that they observed. Instead, they attributed psychological symptoms to “personality disorders” and “routine stressors of confinement”. Moreover, psychotic symptoms such as hallucinations did not prompt consideration of abusive treatment. Importantly, psychological assessments conducted by non-governmental experts revealed diagnostic criteria for current major depression and/or post-traumatic stress disorder (PTSD, a common outcome of torture or ill treatment) in all the cases.

**What Do These Findings Mean?:**

These findings indicate that health professionals assigned to the DoD to provide medical and mental health care to GTMO detainees neglected and/or concealed evidence of intentional harm. Because only nine cases are included in this case series, these findings may not be generalizable to other GTMO detainees. The findings are also limited by their reliance on medical records and case files that were sometimes heavily edited and on psychological assessments based on questionnaires rather than on direct examination. Nevertheless, these findings reveal new information about the potential extent of medical complicity in US torture practices, and they highlight the need for a thorough and impartial investigation of all the available information, including relevant classified information.

**Additional Information:**

Please access these Web sites via the online version of this summary at http://dx.doi.org/10.1371/journal.pmed.pmed.1001027.

- This study is further discussed in the April 2011 *PLoS Medicine* Editorial
- Wikipedia has pages on torture, and on the Guantánamo Bay detention camp note that Wikipedia is a free online encyclopedia that anyone can edit; available in several languages)
- The Office of the UN High Commissioner for Human Rights provides information about the UN Convention against Torture and other Cruel, Inhuman or Degrading Treatment or Punishment and the UN Committee against Torture (in several languages)
- Physicians for Human Rights is a non-profit organization that mobilizes health professionals to advance health, dignity and justice, and promotes the right to health for all. Its Campaign against Torture seeks to restore the US commitment against torture
- Amnesty International provides information about the Guantánamo Bay detention camp

## Introduction

Despite international recognition of torture as a crime that can never be justified [1],[2], the United States government in August 2002 redefined acts such as “waterboarding” (simulated drowning), forced nudity, sleep deprivation, temperature extremes, stress positions, and prolonged isolation to be “safe, legal, ethical, and effective” “enhanced interrogation” techniques (EITs) after the September 11, 2001 attacks on the US. Before then, each of these techniques, alone, was considered to constitute torture by the UN Committee Against Torture and/or the UN Special Rapporteur on Torture [3]. The US recognized them as such in other countries in its country reports on human rights practices [4].

Recent release of previously classified US documents [5]–[8] and non-governmental publications [9]–[11] have demonstrated that physicians and other medical personnel played a critical role in the design and implementation of US torture practices. In the “Bybee memo,” lawyers at the US Department of Justice's Office of Legal Counsel (OLC) set legal thresholds of severe physical and severe and prolonged (“months and even years”) mental pain for torture [12] and required medical monitoring of every application of enhanced interrogation techniques (EITs), ostensibly to ensure that the newly established pain thresholds for torture were not exceeded. The US Central Intelligence Agency (CIA)[13] and Department of Defense (DoD) subsequently established guidelines [14] and standard operating procedures, respectively, for the monitoring of all EITs. These monitoring guidelines, however, did not include any assessment of psychological harm as defined by OLC lawyers [11]. In fact, declassified documents that refer to psychological assessments of detainees indicate that these assessments were conducted by medical personnel to identify psychological vulnerabilities instead of possible evidence of intentional harm [6],[14]. The presence of “non-clinical” medical personnel in intentionally harmful interrogation practices thus enabled the routine practice of ill treatment and torture [9]–[11] in violation of international law [1],[2] and accepted standards of medical ethics [15].

The CIA's Office of Medical Service (OMS) and the DoD's Behavioral Science Consultant Teams (BSCT) designated “non-clinical” health professionals to monitor EITs. Their active roles in acts of torture and ill treatment have been well documented [5]–[11]. Little is known, however, about the role of US Naval Station Guantánamo Bay, Cuba (GTMO) DoD health providers who were responsible for the medical and mental health care of the detainees. These clinical health providers should have been in a position to observe and document physical and psychological evidence of torture and ill treatment.

In this case series, we reviewed GTMO medical records and relevant case files of nine detainees for evidence of ill treatment and torture and assessed the documentation by medical personnel.

## Methods

As non-governmental medical experts retained by legal representatives of GTMO detainees alleging torture and ill treatment, we reviewed a series of nine GTMO medical records, client affidavits, attorney–client notes and summaries, and legal declarations of non-governmental medical experts who were retained by the detainees' attorneys. The legal declarations of non-governmental medical experts were filed in civilian or military court cases. A total of five non-governmental medical experts, including the authors, consulted on behalf of the nine detainees, only one non-governmental medical expert was partially financially compensated for his services. Psychological evaluations were conducted either in-person by the authors (two cases in GTMO), by Physicians for Human Rights (PHR) consultants (one case following release from GTMO), or by proxy evaluations (six cases) developed by a forensic psychiatrist and former DoD consultant who was retained as a medical expert by the detainees' legal representatives. The proxy evaluation included 22 questions on alleged trauma, 37 questions to assess symptoms of post-traumatic stress disorder (PTSD), depression, and anxiety, and a 24-item checklist to assess mental status. In one unclassified case, redacted interrogation plans and interrogation summaries were available as well. Classified information that was available to the authors is not included in this case review.

In all cases, written permission by means of a consent form through legal representatives was provided for the review of the relevant records and possible publication of de-identified case information. After cases were reviewed and findings identified that were thought to merit submission for publication, the study was also reviewed and approved by the Ethics Review Board of PHR. All in-person medical evaluations of torture and ill treatment were conducted in accordance with Istanbul Protocol guidelines [16].

## Results

All of the detainees were incarcerated in GTMO in 2002 with an average incarceration period of seven years. At the time of incarceration, the average age of all nine male detainees was 33.

### Allegations of Torture and Ill Treatment

All of the GTMO detainees alleged torture and ill treatment, inflicted over a period of at least several months and, in some cases, several years, to their attorneys and to non-governmental medical experts (see Table 1). The detainees reported being exposed to an average of eight different forms of EITs (range: five to 11 forms of abuse) including sleep deprivation, temperature extremes, serious threats, forced positions, beating, and forced nudity. In addition to the use of authorized EITs, each of the nine detainees reported being subjected to “unauthorized” acts or torture including: severe beatings, often associated with loss of consciousness and/or bone fractures, sexual assault and/or the threat of rape, mock execution, mock disappearance, and near asphyxiation from water (i.e., hose forced into the detainee's mouth) or being choked. Other allegations included forcing the detainee's head into the toilet, being used as a human sponge to wipe the floor, and desecration of the Quran (e.g., writing profane words in the Quran, stepping on the Quran, and placing it on the floor near the trash). Five of the detainees reported loss of consciousness during interrogation. Seven of the nine detainees reported participating in one or more hunger strikes to protest conditions of detention, and two detainees reported being restrained and forced to receive intravenous fluids and nasogastric tube feedings.

**Table 1:** Allegations of torture and ill treatment (N = 9).

Allegation of Torture/Ill Treatment	No. (%)
Sleep deprivation^a^	9 (100)
Temperature extremes^a^	8 (89)
Serious threats^a^	8 (89)
Forced positions^a^	8 (89)
Beating^a^	8 (89)
Forced nudity^a^	7 (78)
Hooding/sensory deprivations^a^	7 (78)
Prolonged isolation^a^	6 (67)
Religious exploitation^b^	6 (67)
Threatening dogs	6 (67)
Withholding food and/or water^a^	5 (56)
Sexual molestation and/or assault^a^	3 (33)
Mock execution or disappearance^a^	2 (22)
Other forms of ill treatment^c^	9 (100)

a Forms of abuse recognized as torture by the US government prior to 2002.

b Writing curse words in detainee's Quran, throwing detainee's Quran on the floor, stepping on detainee's Quran, religious insults.

c Examples include water hose forced in mouth, pepper spray in clothing, head forced into toilet, dragged on the floor like a “human sponge,” shown pornographic material, spitting in detainees food, farting in detainee's face, not allowing access to toilet, not allowing religious prayer.

### Review of GTMO Medical Records

#### Medical problems

The GTMO medical records indicate that detainees were evaluated and treated on multiple occasions by DoD health providers for a wide range of medical problems unrelated to allegations of abuse, for example: skin rashes, weight loss, diarrhea, low back pain, hemorrhoids, peptic ulcer disease, upper respiratory infections, gingivitis, ear wax removal, and refraction for eye glasses. The average number of medical problems among the detainees was nine with a range of five to 12. In general, the quality of medical documentation for these problems appeared adequate.

#### Physical injuries

In three of the nine cases, the GTMO medical records documented injuries that were consistent or highly consistent with detainee allegations of abuse: contusions (2), bone fractures (3), lacerations (2), peripheral nerve damage (1), and sciatica (2). There was no mention of any cause for these injuries.

Several detainees indicated that access to medical care was linked to cooperation with the interrogators. In one case, a detainee was treated for a painful ankle injury two weeks after the alleged injury. In another case, medical personnel allegedly “certified” the detainee's “fitness” to continue being interrogated after several periods of unconsciousness. Another detainee indicated that he observed interrogators with his medical records and that his chronic back pain was exploited by interrogators with the use of prolonged, painful stress positions.

#### Psychological problems

The medical records indicate that, prior to detention in GTMO, none of the detainees had any past psychological history or family history of psychological problems. GTMO medical personnel documented significant psychological symptoms, however, among eight of the detainees including: nightmares (5), suicidal ideation (4), depression (2), audiovisual hallucinations (3), suicide attempts (2), anxiety/claustrophobia (2), memory and concentration difficulties (1), and dissociative states (2). In each case, the onset of psychological symptoms was temporally related to allegations of abuse and corroborating medical information in the medical records. According to the GTMO medical records, DoD mental health providers with the Behavioral Health Service (BHS) evaluated six of the nine detainees and diagnosed the following: depression (4), passive aggressive personality (4), borderline personality (2), adjustment disorder (3), routine stressors of confinement (2), narcissistic traits (1), psychosis or depression with psychotic features (2), and anxiety NOS (not otherwise specified) (2). Although BHS notes indicated that seven of the detainees had symptoms supporting a diagnosis of PTSD (nightmares, dissociation, memory and concentration difficulties), BHS clinicians did not indicate inquiring about or documenting possible causes of these symptoms and/or the diagnosis of PTSD. Treatment for depression consisted of medications and periodic checks for suicidal/homicidal ideation. In one case, BHS clinical notes document a detainee's symptoms of nightmares, lapses in memory, decreased concentration and appetite, depressed mood, and suicidal thoughts. The medical records indicate that he was treated with antidepressants and told, “[You]…need to relax when guards are being more aggressive.”

Nearly all BHS visits were initiated after suicide attempts and/or detainee hunger strikes. Under these circumstances, BHS notes indicate that the visits were unwelcome and the detainees often refused to cooperate.

### Medicolegal Assessments by Non-Governmental Medical Experts

The assessments conducted by non-governmental medical experts in each of the nine cases indicate that the specific allegations of torture and ill treatment were highly consistent with and supported by physical and psychological evidence observed in all cases. The psychological component of the evaluation revealed diagnostic criteria for current major depression and/or PTSD in all nine cases. Many of the psychological symptoms noted were content-specific for the alleged torture and ill treatment, i.e., nightmares, avoidance behaviors, and triggers for hyperarousal symptoms and exaggerated startle responses.

There was no evidence of malingering or deception by the detainees in any of the evaluations. Each of the detainees provided highly consistent accounts of alleged torture and ill treatment. In one case, unclassified interrogation plans and interrogation summaries provided precise corroboration of the methods of torture and ill treatment that was alleged. There was no evidence of over-endorsement of physical or psychological symptoms and it was clear during the in-person interviews that the detainees' observed affect was internally consistent with the content of the evaluation, for example sadness and crying in the course of recounting shameful experiences such as sexual assault and the expression of anger when describing threats against family members.

Review of one detainee's declassified interrogation plans indicated that BSCT psychologists identified the detainee's psychological and social vulnerabilities; they monitored his interrogations and advised interrogators on how to achieve the ultimate goal of breaking him down psychologically. At one point, the detainee was observed by an interrogator to be having auditory hallucinations in response to extreme sleep deprivation and other abuses. Case documents indicate that a BSCT psychologist was informed of the hallucinations and did nothing to mitigate obvious and profound psychological harm that he/she was made aware of.

## Discussion

The findings of this study demonstrate that allegations by these nine detainees of torture were corroborated by forensic evaluations by non-governmental medical experts and that DoD medical and mental health providers at GTMO failed to document physical and/or psychological evidence of intentional harm.

In each case we reviewed, detainees alleged forms of abuse that are highly consistent with torture as defined by the UN Convention Against Torture as well as the more restrictive US definition of torture that was operational at the time [12]. In one case, unclassified interrogation plans and interrogation summaries provided precise corroboration of the methods of torture and ill treatment that the detainee alleged.

The “enhanced interrogation” techniques that the detainees reported were authorized and implemented by the US in at least three theaters of operation, including GTMO. These acts have been recognized historically as torture and decades of literature have demonstrated the severe physical and mental health consequences of EITs [3],[10],[16]. Legal sources and trained interrogation experts had warned governmental authorities on the questionable legality of EITs and the unreliability of coerced confessions [5]. It appears that the authorization and routine implementation of EITs may have facilitated a command environment in which unauthorized acts of torture were practiced and condoned [9]. This case series of medical evaluations by non-governmental medical experts corroborated the allegations of all detainees of unauthorized acts of torture including, sexual assault, severe beatings, threats of harm to family members, and mock execution and disappearance.

The medical affidavits in each of the nine cases indicate that the specific allegations of torture and ill treatment are highly consistent with physical and psychological evidence documented in the medical records and evaluations by non-governmental medical experts. Using international standards [16] for the medical documentation of torture and ill treatment, non-governmental medical experts were able to correlate physical evidence such as bone fractures, lacerations, contusions, nerve injuries, and psychological evidence, such as symptoms of PTSD and depression—sustained over a period of “months and even years”—with the detainees' allegations of abuse.

The medical evaluations in this case series revealed evidence of severe physical and severe and prolonged psychological pain as stipulated in the Bybee definition of torture. But, according to the Bybee definition of torture, even if the requisite pain thresholds had been exceeded, the infliction of such pain had to be the interrogator's “precise objective” to constitute torture. The condition of “specific intent” for torture is not only inconsistent with international law [1], it undermines the value of medical evidence in the process of justice and accountability. If such “specific intent” was required of perpetrators of domestic violence and/or sexual assault, medical evidence of such crimes would be meaningless unless there was evidence that the perpetrator specifically intended the harms he/she inflicted. While determinations of “specific intent” to commit acts of torture is beyond the scope of this case series, it is clear from a limited number of declassified interrogation logs [12],[17] that the objectives of interrogation included inducing states of “debility,” “dependence,” and “dread,” euphemistically referred to in interrogation logs as, respectively, “ego down,” “futility” and “fear up harsh” [12],[17]. Declassification of interrogation logs and documentation by BSCT and OMS personnel who monitored interrogations would shed considerable light on the intent and imputed intent of interrogators and medical monitors.

The medical doctors and mental health personnel who treated the detainees at GTMO failed to inquire and/or document causes of the physical injuries and psychological symptoms they observed. Psychological symptoms were commonly attributed to “personality disorders” and “routine stressors of confinement.” Temporary psychotic symptoms and hallucinations did not prompt consideration of abusive treatment.

The documentation of torture and ill treatment in medicolegal evaluations conducted by non-governmental medical experts indicates that each of the detainees continues to experience severe, long-term and debilitating psychological symptoms that are likely to persist for many years, and possibly a lifetime.

The failure of “non-clinical” medical monitors of interrogations and DoD clinical health providers to document medical evidence of torture is not surprising. OLC attorneys Jay Bybee and John Yoo attempted in 131 pages of legal memos [12],[18] to transform acts of torture into “safe, legal, ethical, and effective” EITs with the aid of medical monitoring, but they and other policy makers failed to include any meaningful provisions to detect medical evidence of torture as defined by them. The CIA's OMS personnel were required to monitor all EIT practices [13], but had no guidelines for any form of psychological assessment. Similarly, the 2003 and 2004 standard operating procedures [14] for DoD BSCT psychologists indicate the duty to monitor EITs to ensure that they were “safe, legal, ethical, and effective,” but there is no mention of the duty to document abuse until 2005 [14]. Standard operating procedures for DoD health providers also make no mention of documenting abuse until 2005 [19], well after the release of Abu Ghraib photos in 2004 depicting inhumane treatment of detainees in US custody.

The commission and/or concealment of acts of torture should never be justified by any health professionals—clinical, non-clinical, military, or non-military. As the Declaration of Tokyo states “The physician's fundamental role is to alleviate the distress of his or her fellow human beings, and no motive, whether personal, collective or political, shall prevail against this higher purpose” [15].

The findings of this case series indicate that policy makers did not act in “good faith” to ensure “safe, legal, ethical, and effective” EITs as they have claimed [12],[18]. In reality, the implementation of EITs included “unauthorized” acts of torture, were inflicted over prolonged periods of time, and resulted in severe and prolonged physical and mental pain. The abuses reported in this case series could not be practiced without the interrogators and medical monitors being aware of the severe and prolonged physical and mental pain that they caused.

### Limitations

The cases selected for review included only those for which the authors were consulted. The findings included in this commentary may not be generalizable to other GTMO detainees. The findings are based on medical records and case files which, in some cases, are heavily redacted. Also, psychological evaluations in the majority of cases consisted of proxy assessments. Despite these limitations, non-governmental medical evaluators had sufficient information to form their medicolegal assessments and to qualify any uncertainties related to limited access to case information.

### Conclusion

The findings in these nine cases indicate that medical doctors and mental health personnel assigned to the US Department of Defense neglected and/or concealed medical evidence of intentional harm. The full extent of medical complicity in US torture practices will not be known until there is a thorough, impartial investigation including relevant classified information. We believe that, until such time as such an investigation is undertaken, and those responsible for torture are held accountable, the ethical integrity of medical and other healing professions remains compromised.

## Abbreviations

BSCT: DoD Behavioral Science Consultant Teams
CIA: US Central Intelligence Agency
DoD: US Department of Defense
EIT: enhanced interrogation technique
GTMO: U.S. Naval Station Guantánamo Bay, Cuba
OLC: DoD’s Office of Legal Counsel
OMS: CIA Office of Medical Service
PHR: Physicians for Human Rights
PTSD: post-traumatic stress disorder
